# Genetic testing and personalized ovarian cancer screening: a survey of public attitudes

**DOI:** 10.1186/s12905-016-0325-3

**Published:** 2016-07-26

**Authors:** Susanne F. Meisel, Belinda Rahman, Lucy Side, Lindsay Fraser, Sue Gessler, Anne Lanceley, Jane Wardle, for the PROMISE-2016 study team

**Affiliations:** 1 Department of Epidemiology and Public Health, Health Behaviour Research Centre, University College London, Gower Street, London, WC1E 6BT UK; 2 Department of Women’s Cancer, EGA UCL Institute for Women’s Health, University College London, London, UK

**Keywords:** Stratification, Breast cancer, Ovarian cancer, Predictive genetic testing, Risk, Implementation

## Abstract

**Background:**

Advances in genetic technologies are expected to make population-wide genetic testing feasible. This could provide a basis for risk stratified cancer screening; but acceptability in the target populations has not been explored.

**Methods:**

We assessed attitudes to risk-stratified ovarian cancer (OC) screening based on prior genetic risk assessment using a survey design. Home-based interviews were carried out by the UK Office of National Statistics in a population-based sample of 1095 women aged 18–74. Demographic and personal correlates of attitudes to risk-stratified OC screening based on prior genetic risk assessment were determined using univariate analyses and adjusted logistic regression models.

**Results:**

Full data on the key analytic questions were available for 829 respondents (mean age 46 years; 27 % ‘university educated’; 93 % ‘White’). Relatively few respondents felt they were at ‘higher’ or ‘much higher’ risk of OC than other women of their age group (7.4 %, *n* = 61). Most women (85 %) said they would ‘probably’ or ‘definitely’ take up OC genetic testing; which increased to 88 % if the test also informed about breast cancer risk. Almost all women (92 %) thought they would ‘probably’ or ‘definitely’ participate in risk-stratified OC screening. In multivariate logistic regression models, university level education was associated with lower anticipated uptake of genetic testing (*p* = 0.009), but with more positive attitudes toward risk-stratified screening (*p* <0.001). Perceived risk was not significantly associated with any of the outcome variables.

**Conclusions:**

These findings give confidence in taking forward research on integration of novel genomic technologies into mainstream healthcare.

## Background

Although ovarian cancer is one of the leading causes of cancer death in women [1], population-based screening is not currently recommended because reliable early risk-markers have not yet been identified [2–4]. For example, the Prostate, Lung, Colorectal and Ovarian (PLCO) Cancer Screening Trial demonstrated that current tests (transvaginal ultrasound and CA-125) aimed at detecting ovarian cancer early enough to reduce mortality were ineffective. Further, results from this trial showed that current risk profiling methods did not result in reduced OC mortality when applied retrospectively to PLCO [5]. However, improved discrimination on the basis of genetic predisposition to ovarian cancer, alongside increasing affordability of genome sequencing, may improve identification of ‘at-risk’ individuals. Focusing screening on women at the highest genetic risk would avoid the risk of false positives for those at lower risk, and could make ovarian cancer screening feasible and cost-effective in the near future [6].

Currently, efforts are under way to develop and validate models for risk stratification, early detection and diagnosis of OC which incorporate clinical, epidemiological, proteomic and genetic data (PROMISE 2016 ‘Predicting Risk of Ovarian Malignancies, Improved Screening and Early Detection’). Genetic models will include high penetrance genes associated with hereditary breast and ovarian cancer, BRCA1 and BRCA2, as well as lower penetrance genes associated with ovarian cancer susceptibility BRIP1, RAD51C and RAD51D [7–9]. Given the 10 % risk of ovarian cancer associated with Lynch syndrome [10], mismatch repair genes MLH1, MSH2, MSH6 and PMS2 will be included. The model could be modified should other relevant genes be identified. The model will also include clinical and epidemiological data (family history, environmental, hormonal and reproductive factors). A workstream dedicated to proteomics aims to identify novel early indicators of OC to improve on current predictive values of CA-125. Women participating in the programme will receive an estimation of OC risk (low, intermediate or high). Women will be offered risk stratified information, support and clinical intervention (screening or surgery) depending on their personal risk.

However, the success of any cancer screening programme depends on its acceptability in the target population [11]. Given that early research indicates that population-based genetic testing may improve current family history based risk prediction approaches in a cost-effective manner [12–14] it is timely to assess public attitudes on this topic. Negative attitudes towards population-based genetic testing would render the genetic stratification approach unsuitable for existing or new cancer screening programmes. Few studies have investigated attitudes to genetic testing for cancer risk in general population samples. A review of studies carried out in the United States found positive attitudes towards predictive genetic testing for cancer, with anticipated uptake of testing of over 70 %; although actual uptake has been found to be somewhat lower [15]. A qualitative study in the UK investigated attitudes to risk-stratified ovarian cancer screening that incorporated genetic risk assessment, and found that women were very positive about it [16]. Similarly, a qualitative study in the Netherlands reported positive attitudes towards amending the current breast cancer screening programme to include risk-stratification based on genetic risk; with the proviso that women who wanted to access screening despite having low genetic risk could still do so [17]. However, because these samples were small, the findings cannot be generalised to the wider population.

Research investigating the predictors of interest in cancer genetic testing has predominantly focused on individuals with a strong family history of cancer. In these studies, age, ethnicity and perceived risk have frequently been shown to be associated with higher interest in testing [15]. However, because of the psychological and health implications of a strong family history of cancer, these results cannot be assumed to generalise to the wider population.

The aims of the present study were therefore to explore public attitudes of women in the UK towards risk-stratified screening based on prior genetic risk assessment for OC, and identify demographic and personal predictors of anticipated uptake of both genetic testing and screening.

## Methods and procedure

### Sample

Data were collected as a part of the Office of National Statistics (ONS) ‘Opinions and Lifestyle’ survey in January and March 2014. This is a population-based survey of adults aged 16–94 years conducted by the ONS on behalf of government departments, non-governmental agencies, and academic institutions. The sample covers Great Britain (England, Wales and Scotland), excluding the Isles of Scilly and the Scottish Highlands and Islands. Modules include a range of topics; minimizing the risk of respondent bias for specific topics. Respondents are given the option to withdraw at any time. Each month, households (*n* = 2010) are identified from the Royal Mail’s Postcode Address File using stratified random probability sampling. The selected addresses are contacted up to eight times at different times and days of the week to maximize response rates. One person aged over 16 from each household is randomly chosen to complete a computer-assisted, face-to-face interview with a trained interviewer. Verbal informed consent was obtained from participants before commencing the survey. Since this study was a population-based anonymous survey, it was exempt from ethical approval, as per guidelines set out by the UCL Ethics committee for non-NHS research (http://ethics.grad.ucl.ac.uk/exemptions.php).

The genetic testing/screening module for this study was introduced with a brief statement about the questions and a brief introduction to the topic: *‘The next set of questions is about genes and cancer. The first questions are about genes. Genes contain the ‘instruction manual’ of life, called DNA. Genes are passed from parents to their children. Nowadays, it is possible to predict whether someone is likely to develop certain diseases by looking at their genes. This is called genetic testing.’*

### Measures

#### Outcome variables

The attitude items were specifically developed for this survey. The questions were read aloud, but we used the Flesch Reading Ease formula and the Flesch-Kincaid Grade Level formula to provide a formal estimate of comprehension (https://readability-score.com/). This produced a total score of 68 for all questions, which translates to the reading level expected in grade 6 (age 12).

Three questions addressed anticipated uptake of genetic testing in the National Health Service (NHS). The NHS is the provider of healthcare in the UK. The NHS runs all the organised cancer screening programmes. Response options for all questions were: *‘no, definitely not’; ‘no probably not’; ‘yes, probably’* and *‘yes, definitely’*.

- *‘If the NHS offered a genetic test for the risk of ovarian cancer, would you take up the offer’?*
- *‘If the genetic test for ovarian cancer also told you about your risk of breast cancer, would you take up the offer’?*
- ‘*A small number of women may be found to be at very high risk when they have the genetic test. They would be offered surgery to have their ovaries removed. Considering that your genetic test result might mean you are offered surgery to remove your ovaries, would you still take up the offer of a genetic test’?*

Two questions addressed attitudes to risk-stratified ovarian cancer screening:

- *‘If you were invited for NHS screening for ovarian cancer, would you take up the offer’* (*‘no, definitely not’; ‘no probably not’; ‘yes, probably’; ‘yes, definitely’*)?
- *‘It’s possible that women who are found to be at higher genetic risk will be offered more frequent screening for ovarian cancer, and those found to be at lower risk will be offered less frequent screening. What do you think of the idea of varying the frequency of ovarian screening’* (*‘very bad idea’; ‘bad idea’; ‘not sure’; ‘good idea’ and ‘very good idea’)?*

Perceived relative risk of ovarian cancer was assessed with one question: ‘*Compared with other women of your age, what do you think are your chances of getting ovarian cancer*’? Response options were: *‘much lower than others’*, *‘lower than others’, ‘the same as others’, ‘higher than others’*, *‘much higher than others’*. For the current analyses, the first three and last two categories were combined to reflect lower/average vs. higher perceived risk.

Demographic data were provided by ONS from their standard survey items (http://www.ons.gov.uk/ons/index.html). Age was included as a continuous variable in statistical analyses. Ethnicity was classified as ‘White’ vs. ‘Ethnic minority’ because the individual ethnic minority sub-groups were small; making subdivision inappropriate. Educational attainment was classified as university degree or equivalent vs. below degree level. Marital status was coded as married/cohabiting vs. single/widowed/divorced.

### Statistical analyses

We only included women age 18–74 years to reflect the population to whom ovarian cancer screening might be offered. We excluded participants who had missing data for any of the variables in the analyses.

Statistical analyses were carried out using the Statistical Package for Social Sciences (SPSS) version 20.0 (SPPS Inc., Chicago, IL). Univariate χ^2^ analyses were used to explore demographic predictors and the impact of perceived risk on each dichotomous outcome variable. Multivariate logistic regression was used to establish the associations with: i) the three items on anticipated uptake of genetic testing, and ii) the two items on attitudes to risk-stratified screening. The first model included only demographic characteristics, and the second one added perceived risk. Bonferroni corrections were used to correct for multiple testing, so α was set at 0.05/5 = 0.01.

## Results

Data for the current study were collected in two waves: January and March 2014. In the January wave, 8 % (*n* = 166) of the 2010 selected households were not eligible because they were businesses or empty properties. Of 1844 eligible households, 9 % (*n* = 171) could not be contacted and 33 % (*n* = 608) declined to take part in the ONS survey. In the March wave, 8 % (165) of selected households were not eligible because they were business or empty properties. Of the 1853 eligible households, 13 % (*n* = 237) could not be contacted and 31 % (*n* = 578) chose not to take part. Therefore, the overall response rate was 57 %, comparable to previous ONS surveys (http://tinyurl.com/z8apjt6). The total female sample was *n* = 1095.

### Sample characteristics

After exclusion of individuals who were ineligible due to age (*n* = 138) or missing data (*n* = 128), the final sample for analysis consisted of 829 women aged 18–74 (mean 46 years; SD = 15.5 years). Most were ‘White’ (92.9 %, *n* = 770), just over half were married or cohabiting (54.8 %, *n* = 454), and just over a quarter (27.3 %, *n* = 226) were at least university educated. With the exception of education, which was higher in this sample, demographic characteristics were comparable to the UK female population in this age group (ONS Census 2011: http://www.ons.gov.uk/ons/guide-method/census/2011/uk-census/uk-census-data-releases/index.html).

### Anticipated uptake of genetic testing for ovarian cancer

Overall, interest in genetic testing for ovarian cancer was very high. Most women said they would ‘probably’ or ‘definitely’ take up an offer of genetic testing (84.9 %, *n* = 734), which increased to 88.3 % (*n* = 819) if the test also informed about breast cancer risk. Anticipated ‘probable’ or ‘definite’ uptake remained high (85.7 %, *n* = 787) if the test result indicated they were at very high risk and the appropriate risk management recommendation was risk-reducing surgery.

There were few demographic differences in anticipated uptake of genetic testing for ovarian cancer risk (Table 1). Ethnic minority status was associated with lower anticipated uptake of genetic testing only if it involved a potential offer of surgery in univariate analyses. Table 2 shows that the association remained significant in Bonferroni corrected logistic regression models after adjusting for all other demographic and personal variables (OR = 2.14, 95 % CI = 1.25-3.66, *p* = 0.006). University level education was not associated with anticipated uptake of genetic testing in univariate analyses, but it became significantly associated with lower anticipated uptake once demographic and personal factors were taken into account (OR = 0.67, 95 % CI = 0.49-0.94, *p* = 0.009).

**Table 1:** Univariate analyses of anticipated uptake of genetic testing for ovarian cancer risk

Variable	Probable/definite uptake of OC genetic testing if offered by NHS	Probable/definite uptake of OC genetic testing if it also informs about BC risk	Probable/definite uptake of OC genetic testing with potential for surgery	Probable/definite uptake of OC screening	Good/very good idea to vary frequency of OC screening by risk
% Total (*N* = 829)	84.1	88.1	85.6	92.3	66.6
Mean Age (SD)	46.9 (15.6)	46.6 (15.2)	48.1 (15.1)	46.9 (15.2)	46.8 (15.4)
Ethnicity					
% Ethnic minority	85.2	83.6	72.6**	91.8	59.0
% White	84.0	88.6	86.5	92.6	67.3
Education					
% Below university	84.3	86.9*	84.5	91.6	62.4**
% University	84.0	92.0	89.0	95.2	78.6
Marital status					
% Married/cohabiting	85.0	89.6	83.9	93.6	68.3
% Single/widowed/divorced	83.3	86.7	87.3	91.4	65.0
Perceived risk of ovarian cancer					
% Much lower/lower/the same as others	84.1	88.0	84.9	92.6	67.3
% Higher/much higher than others	85.7	90.5	90.2	92.1	69.8

*Abbreviations*: *OC* ovarian cancer, *BC* breast cancer, *NHS* National Health Service, the provider of healthcare and organised screening in the UK; * *p* <0.05; ** *p* <0.01

**Table 2:** Multivariate logistic regression of demographic predictors of anticipated uptake of genetic testing for ovarian cancer risk

Variable	Probable/definite uptake of OC genetic testing if offered by NHS			Probable/definite uptake of OC genetic testing if it also informs about BC risk			Probable/definite uptake of OC genetic testing with potential for surgery			Probable/definite uptake of OC screening if offered by the NHS			Good/very good idea varying frequency of OC cancer screening by personal risk		
N = 829	OR	95 % CI	p-value	OR	95 % CI	p-value	OR	95 % CI	p-value	OR	95 % CI	p-value	OR	95 % CI	p-value
Age (trend)	0.99	0.98-1.02	0.153	0.99	0.98-1.00	0.081	1.01	1.00-1.01	0.028	1.00	0.99-1.00	0.924	1.00	0.99-1.01	0.881
Ethnicity															
Ethnic minority	1			1			**1**			1			1		
White	1.67	0.98-2.83	0.057	1.62	0.95-2.75	0.073	**2.14**	**1.25-3.66**	**0.006**	1.85	1.06-3.23	0.028	1.51	0.87-2.60	0.136
Education															
Below university	**1**			1			1			1			**1**		
University	**0.67**	**0.49-0.94**	**0.009**	0.76	0.56-1.03	0.078	0.93	0.69-1.27	0.938	1.15	0.88-1.79	0.204	**2.22**	**1.57-3.14**	**<0.001**
Marital status															
Sgl/wid/div	1			1			1			1			1		
Married/cohab	1.24	0.95-1.62	0.103	1.06	0.80-1.39	0.664	1.41	1.08-1.84	0.012	1.35	1.00-1.83	0.050	1.08	0.82-1.43	0.568

*Abbreviations*: *Sgl* single, *wid* widowed, *div* divorced, *cohab* cohabiting

Analyses are mutually adjusted for age (modelled as continuous variable), ethnicity, education and marital status (modelled as binary variables)

BOLD significant after adjustment for multiple testing *p* < 0.01

### The impact of perceived personal risk on anticipated uptake of genetic testing

Relatively few participants felt that they were at ‘higher’ or ‘much higher’ risk of ovarian cancer than other women of their age group (7.4 %, *n* = 61). Perceived risk was not significantly associated with anticipated uptake of ovarian cancer genetic testing in univariate analyses (*p* = 0.729), and adding it to the multivariate model made no difference to the other associations (data not shown).

### Anticipated uptake of, and attitudes to, risk-stratified screening for ovarian cancer

Anticipated uptake of ovarian cancer screening was also very high, with 92.3 % of women reporting that they would ‘probably’ or ‘definitely’ take up ovarian cancer screening if invited. Over two thirds of the sample would support risk-stratified ovarian cancer screening (66.6 %).

There were few demographic associations with anticipated uptake of ovarian screening or attitudes to risk-stratified screening. University level education was associated with more positive attitudes towards risk-stratified screening in univariate and multivariate analyses (multivariate OR = 2.22, 95 % CI = 1.57-3.14, *p* <0.001).

### The impact of perceived risk on anticipated uptake of, and attitudes to, risk-stratified screening for ovarian cancer

Perceived risk was not significantly associated with anticipated uptake of ovarian cancer screening in univariate analyses (*p* = 0.882). Slightly more respondents who perceived themselves as higher risk than lower risk supported risk-stratified screening (69.8 % vs. 67.3 %), but the difference was not significant (*p* = 0.685). Perceived risk was also not significantly associated with any of outcomes in multivariate models (data not shown).

## Discussion

This is the first quantitative study of attitudes to population-based genetic testing with subsequent risk-stratified screening for ovarian cancer screening. Although the scenarios used in the survey were somewhat hypothetical since OC screening is currently not recommended, the results show high levels of support for stratified cancer screening based on prior genetic risk assessment, with little variation across demographic and personal characteristics.

Similar to earlier qualitative studies, [16, 17] most survey respondents were enthusiastic about a risk-stratified approach to screening, and over two thirds would support it. However, supporters were more likely to hold a university degree than those more sceptical of risk-stratified screening. It is possible that less educated respondents felt less knowledgeable about the concept of personalized screening, and therefore less certain of benefits and disadvantages. Future work could explore the reasons behind this finding to address any information needs and concerns early on. Age was not associated with attitudes towards risk-stratified screening which suggests that personal experience of breast cancer screening may not overly influence attitudes. However, overall, these results suggest slightly more openness to risk stratified screening than described by Henneman and colleagues [17] in the Dutch sample. It is possible that introduction of a new risk-stratified screening programme is met with less resistance than the prospect of amending an existing cancer screening programme, and this hypothesis could be explored in future research.

Ethnic minority status was associated with less positive attitudes towards OC genetic testing only if it involved the potential offer of surgery to have the ovaries removed. This suggests that factors other than the prospect of genetic testing *per se* may be important contributors to the decision of whether to undergo genetic testing. However, because we had to combine women from all ethnic subgroups because individual subgroups were small – approximating population levels – the current findings should be viewed with caution. Future qualitative research could investigate attitudes towards genetic testing in women from ethnic minority subgroups further.

Although perceived risk has often been described as an important predictor of both anticipated and actual uptake of genetic testing and screening, [18–20] we did not find significant associations with attitudes to genetic testing or risk-stratified screening. Differences in the measure of perceived risk may contribute to the difference; earlier studies assessed absolute risk perception which may result in an overestimation of risk, whereas we assessed perceived relative risk in the current study, which has been shown to be more robust [21, 22]. However, it is also possible that the widespread use of single item measures reduces the reliability of the results, especially where the proportion of women who perceive themselves to be at high risk is low – as was the case in this study.

In the present study interest in genetic testing was very high despite few women perceiving their risk as high. This suggests that women would choose to undergo genetic testing for reasons other than perceived high personal risk of ovarian cancer. One possibility is that they have altruistic motives; wanting to obtain information for their children and families, as suggested by findings from studies concerned with uptake of personal genomic testing [23, 24]. Alternatively, they may want to obtain the information for its own value, or because they self-identify as an information-seeker [25]. Characterising reasons for taking part in genetic testing without significant family history and low perceived risk of ovarian cancer will be important because they may influence subsequent reactions to the test results.

This study had some strengths. The question module was included in a large monthly survey conducted by an external agency (ONS) which reduces the likelihood of response bias related to the specific subject matter. The sample had been recruited by stratified random sampling which is the ‘gold-standard’ of survey recruitment; therefore, it is broadly representative of the UK population of women aged 18–74. We adjusted analyses for multiple testing, which gives confidence in the robustness of the current findings.

The study also had limitations. At this stage, neither population-based genetic testing nor OC screening is recommended. Therefore, all the questions alluded to hypothetical scenarios rather than real testing/screening offers, and these have been found to correlate only modestly with actual behaviour [15, 26]. However, the results are useful for gauging general attitudes towards risk-stratified screening programme in a cost-effective manner. Although the response rate was comparable to other ONS surveys, there are a large proportion of potential respondents whose views are unknown, limiting generalizability of the findings. However, refusal was for the survey in general, and not for the specific module. All questions in this survey were ‘closed’, which prevented us exploring the answers in more detail. Further qualitative research could be useful to examine women’s thinking about population-based genetic testing and risk-stratified screening. Finally, this survey was conducted after the well-known actress Angelina Jolie had publicly discussed her BRCA positive status and risk-reducing bilateral mastectomy, which may have brought genetic testing to the attention of the general population and influenced attitudes [27–29].

Stratified cancer screening based on genetic risk is considerably more complex than simple age-stratified approaches and raises many ethical, legal, social and organisational aspects [30]. Chowdhury and colleagues have outlined these in detail, including issues relating to test sensitivity and specificity, effective risk communication, communication of incidental findings, potential for discrimination, and the need for adequate preparation of the healthcare workforce [31]. Despite these challenges, it is interesting to see that public opinion of women in the UK appears to support efforts to ‘personalize’ cancer screening.

## Conclusion

The findings from this study suggest that women in the UK are ready to accept the introduction of population-based genetic testing for personalisation of future cancer screening programmes; giving confidence in taking forward research on integration of novel genomic technologies into mainstream healthcare.
